# Supplementary material for: Disrupted Coupling of Heart Rate–Dependent Brain Network Switching and Attentional Task Performance in Schizophrenia Spectrum Disorders
Source: medRxiv. 2026 Apr 9:2026.04.06.26350241. Preprint. [Version 2] doi: 10.64898/2026.04.06.26350241 (PMC13086097; doi:10.64898/2026.04.06.26350241)
Supplement: Supplementary file 1 [file NIHPP2026.04.06.26350241v2-supplement-1.pdf]

medRxiv preprint doi: https://doi.org/10.64898/2026.04.06.26350241; this version posted April 9, 2026. The copyright holder for this preprint (which was not certified by peer review) is the author/funder, who has granted medRxiv a license to display the preprint in perpetuity. This article is a US Government work. It is not subject to copyright under 17 USC 105 and is also made available for use under a CC0 license.

Supplementary Information

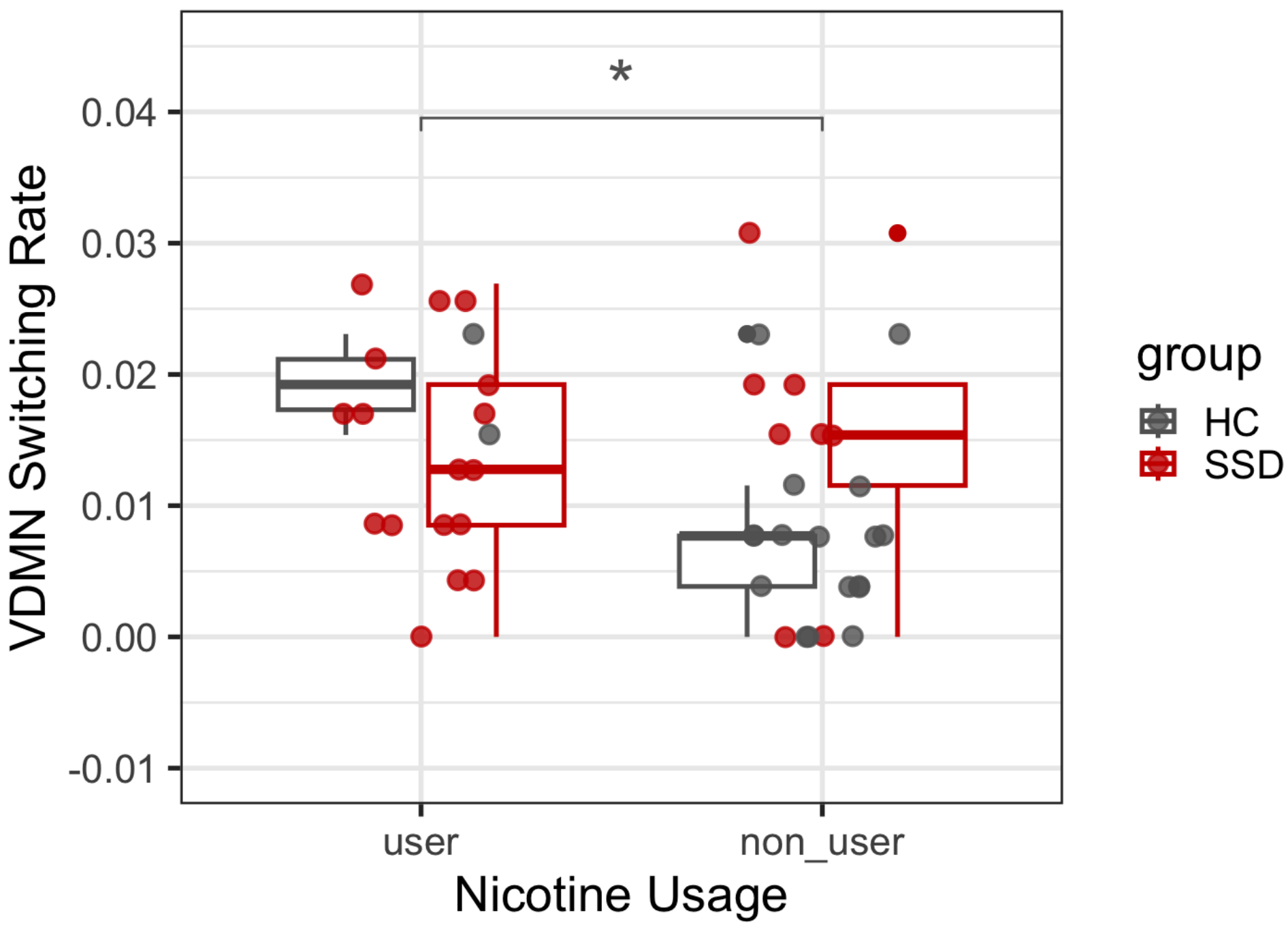

Supplementary Figure 1. VDMN Switching Across Nicotine Users and Non-Users Across Groups. A significant difference was found only in the HC group. (*) p<0.05.
